# Growth, Quality, and Nitrogen Metabolism of *Medicago sativa* Under Continuous Light from Red–Blue–Green LEDs Responded Better to High Nitrogen Concentrations than Under Red–Blue LEDs

**DOI:** 10.3390/ijms252313116

**Published:** 2024-12-06

**Authors:** Ren Chen, Yanqi Chen, Kunming Lin, Yiming Ding, Wenke Liu, Shurong Wang

**Affiliations:** 1 Guangdong-Hong Kong-Macao Joint Laboratory for Intelligent Micro-Nano Optoelectronic Technology, School of Physics and Optoelectronic Engineering, Foshan University, Foshan 528225, China; chenren@fosu.edu.cn (R.C.); china.mollyding@outlook.com (Y.D.); 2 Institute of Environment and Sustainable Development in Agriculture, Chinese Academy of Agricultural Sciences, Beijing 100081, China; cyqmogu@163.com (Y.C.); kunminglin921@163.com (K.L.); 3 Key Lab of Energy Conservation and Waste Management of Agricultural Structures, Ministry of Agriculture and Rural Affairs, Beijing 100081, China

**Keywords:** plant factory with artificial light (PFAL), alfalfa, yield, crude protein, enzyme activity

## Abstract

Alfalfa is a widely grown forage with a high crude protein content. Clarifying the interactions between light quality and nitrogen level on yield and nitrogen metabolism can purposely improve alfalfa productivity in plant factories with artificial light (PFAL). In this study, the growth, quality, and nitrogen metabolism of alfalfa grown in PFAL were investigated using three nitrate-nitrogen concentrations (10, 15, and 20 mM, labeled as N_10_, N_15_, and N_20_) and continuous light (CL) with two light qualities (red–blue and red–blue–green light, labeled as RB-C and RBG-C). The results showed that the adaptation performance of alfalfa to nitrogen concentrations differed under red–blue and red–blue–green CL. Plant height, stem diameter, leaf area, yield, Chl a + b, Chl a, Chl b, crude protein contents, and NiR activity under the RB-CN_15_ treatment were significantly higher than RB-CN_10_ and RB-CN_20_ treatments. The RB-CN_20_ treatment showed morphological damage, such as plant dwarfing and leaf chlorosis, and physiological damage, including the accumulation of proline, H_2_O_2_, and MDA. However, the difference was that under red–blue–green CL, the leaf area, yield, and Chl a + b, carotenoid, nitrate, and glutamate contents under RBG-CN_20_ treatment were significantly higher than in the RBG-CN_10_ and RBG-CN_15_ treatments. Meanwhile, the contents of soluble sugar, starch, and cysteine were significantly lower. However, the crude protein content reached 21.15 mg·g^−1^. The fresh yield, dry yield, stomatal conductance, leaf area, plant height, stem diameter, crude protein, GS, and free amino acids of alfalfa were positively correlated with increased green light. In addition, with the increase in nitrogen concentration, photosynthetic capacity, NiR, and GOGAT activities increased, promoting growth and improving feeding value. The growth, yield, photosynthetic pigments, carbon, nitrogen substances, and enzyme activities of alfalfa were significantly affected by the interaction between nitrogen concentration and light quality, whereas leaf/stem ratio and DPPH had no effect. In conclusion, RB-CN15 and RBG-CN_20_ are suitable for the production of alfalfa in PFAL, and green light can increase the threshold for the nitrogen concentration adaptation of alfalfa.

## 1. Introduction

With the impressive development of China’s dairy and livestock industries, the demand for forage has increased in response to severe protein feed shortages in recent years. Alfalfa (*Medicago sativa* L.), considered the king of pasture, represents a highly adaptable, productive, and protein-rich forage with elevated nutritional value for various livestock [1,2]. To meet the growing demand for forage, alfalfa has been extensively cultivated. At present, China has become the second-largest country in terms of acreage for alfalfa cultivation [3]. However, in arid and semi-arid regions, where the lack of nutrient soil regions of north and northwest China is not conducive to the cultivation of alfalfa, it limits the development of alfalfa pasture [4]. Previous studies have shown that environmental and field management factors, such as precipitation, temperature, light, heavy metal pollution, soil, and nitrogen fertilizer addition, can influence the growth and photosynthesis of alfalfa, resulting in differences in yield and nutrient content [5,6,7]. Therefore, there is an urgent need for a modern production facility that avoids the environmental problems faced by field cultivation, provides an adequate fertilizer supply, and ensures forage with stable yields and quality.

Plant factories with artificial light (PFAL) in a controlled environment provide stable lighting, nutrients, and temperature. They are contemporary horticultural production facilities known for their automation, high production efficiency, optimal land utilization, resilience to weather and pests, and product safety. PFAL have recently been utilized for cultivating leaf vegetables, edible sprouts, medicinal plants, and crops [8,9,10]. They have also shown promise in forage production, especially in the successful production of barley using hydroponics, maize, and wheat seeding as fodder. However, the crude protein content of hydroponically grown forage was low, ranging from 11% to 15% [11,12]. In the production of alfalfa in PFAL, our previous research has obtained a series of conclusions that a light intensity of 400–500 μmol·m^−2^·s^−1^, a harvesting period of 30 days, and a light quality of red–blue–green were optimal light conditions. Moreover, alfalfa was proven to be a plant tolerant to continuous light (CL) [13,14]. In addition, Tang et al., 2022 also found that a light intensity of 400–500 μmol·m^−2^·s^−1^ was conducive to the synthesis of carbohydrates in alfalfa [15]. He et al., 2022 found that an increase in blue light components inhibited the accumulation of dry mass by regulating stomatal conductance [16]. The aforementioned studies have verified the feasibility of using PFAL to produce alfalfa, but research on environmental factors such as temperature, air, and nutrient solutions is still lacking.

Nitrogen is an indispensable element in nutrient solutions that is used in the composition of biological macromolecules such as proteins, enzymes, and nucleotides. Additionally, it acts as a signaling molecule and plays a crucial role in the growth, metabolism, physiology, and development of plants. Nitrogen in the nutrient solution is primarily supplied by nitrate (NO_3_^–^) and ammonium (NH_4_^+^). Nitrate and ammonium account for 70% of anion and cation absorption by plants [17]. The form and amount of nitrogen in the nutrient solution will have a significant impact on growth and metabolism. The nitrogen concentration is also strongly correlated with the content of primary and secondary metabolites, photosynthetic performance, and chlorophyll content of plants [18,19,20]. The nitrate absorbed by plants is assimilated by the key enzymes of nitrogen metabolism, nitrate reductase (NR, EC 1.7.1.3) and nitrite reductase (NiR, EC 1.7.1.3), to produce ammonium. This process is catalyzed by the GOGAT/GS pathway to form glutamate and glutamine for amino acid synthesis [21,22]. Nitrogen metabolism depends on the carbon skeleton and energy synthesized through photosynthesis. The activity of key enzymes has been confirmed to be regulated by the light intensity, light quality, and light mode [23,24].

The synergistic interaction between nitrogen and light affects the growth and development of plants, as well as the absorption and metabolism of nitrogen [22,24,25]. In a study conducted by Wen et al., 2022 on the growth of tall fescue under four treatments involving two light intensities and two nitrogen concentrations, the results showed that a low nitrogen level enhances photosynthesis by balancing carbon and nitrogen metabolism in the roots and leaves, thereby improving tolerance to stress induced by low light levels [24]. Liang et al., 2023 designed three experiments on lettuce cultivation involving different light qualities and two nitrate concentrations [25]. Transcriptional analysis revealed a synergistic effect, where nitrate depletion combined with blue light activated the accumulation of flavonoids in lettuce. Liu et al., 2024 conducted a two-factor three-level orthogonal test to study flavonoid accumulation in lettuce and nitrogen levels in three types of nutrient solutions [22]. Through analyzing biomass, nitrogen metabolism enzyme activity, and gene expression, they discovered a significant synergy between two environmental factors: the proportion of green light in the spectrum and nitrogen nutrient levels in the solution, affecting growth and nitrogen metabolism.

Nitrogen application and the light environment can regulate nitrate absorption and nitrogen metabolism, thus affecting the crude protein content of plants [24]. Alfalfa with a high crude protein content is considered to have a high nutritional value and is valuable for increasing dairy production and quality [26]. Some studies have suggested that there is a specific threshold for nitrogen uptake in alfalfa. Below this threshold, growth can be promoted, while above it, growth is negatively affected, and the quality of feeding is reduced. Zhang et al., 2020 found that when applying the same phosphate fertilizer, the crude protein content of alfalfa in the low-nitrogen treatment was higher than that in the no-nitrogen and high-nitrogen treatments [27]. Kamran et al., 2022 found that the correct application of nitrogen (150 kg·ha^−1^) had a positive promoting effect on increasing alfalfa yield compared to no fertilizer application [5]. Excessive nitrogen application (300 kg·ha^−1^) in alfalfa has been shown to increase greenhouse gas emissions. On the one hand, the excessive application of nitrogen fertilizer can lead to stress [28]. On the other hand, excessive fertilization decreases nitrogen use efficiency and increases nitrogen losses [29].

However, little is known about the nitrogen requirements of alfalfa in soilless cultivation and the appropriate concentration that can meet growth demands without causing high nitrogen stress. The interaction effects of the quality of CL and nitrogen concentrations on the quality and nitrogen metabolism of alfalfa are not clear. Previous studies have shown that under the same light intensity, red–blue–green light can increase leaf area, chlorophyll content, and NR and GS activities compared with red–blue light [14,23]. Therefore, we expected that red–blue–green light could enhance the adaptability of alfalfa to nitrogen concentrations. Although red–blue–green light can improve yield and quality, red–blue light remains the dominant light quality used in PFAL. Therefore, we selected red–blue light and red–blue–green light as light sources to assess suitable nitrogen concentrations. The objective of this study was to investigate whether the interaction of light and nitrogen with CL could promote alfalfa growth, yield, nutrient content, and nitrogen metabolism. The results of this research will help in designing an appropriate light environment and nitrogen concentration in the nutrient solution for producing high-quality forage in PFAL.

## 2. Results

### 2.1. Growth Characteristics and Yield

**Figure 1:**
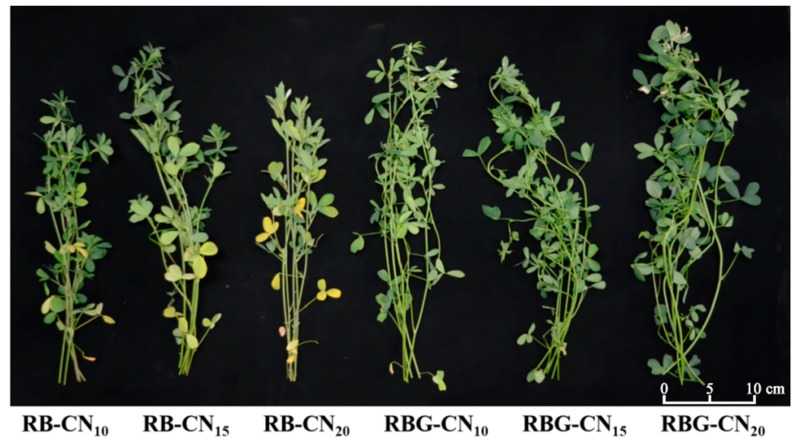
Appearance of alfalfa under three nitrogen concentrations and CL with two light qualities.

The morphology of alfalfa under three different nitrogen concentrations was observed under red–blue and red–blue–green CL (Figure 1). Alfalfa exposed to red–blue CL exhibited a noticeable yellowing of leaves. The plants appeared stunted, with sparse leaves, shedding of basal leaves, and increased leaf chlorosis under the RB-CN_20_ condition. Alfalfa with red–blue–green CL showed a luxuriant plant type, increased leaf number, and leaf areas with an increase in nitrogen concentrations. No withered or yellow leaves were observed.

**Figure 2:**
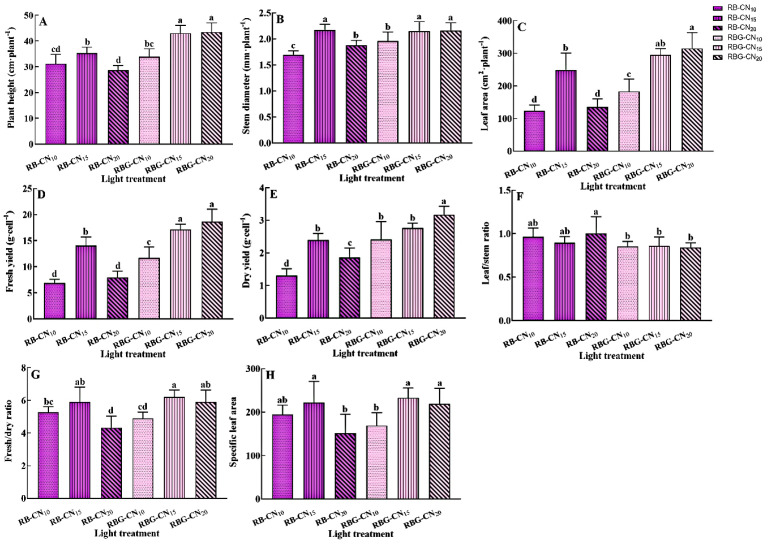
Growth characteristics and yield of alfalfa under three nitrogen concentrations with red–blue and red–blue–green CL. (**A**) Plant height, (**B**) stem diameter, (**C**) leaf area, (**D**) fresh yield, (**E**) dry yield, (**F**) fresh/dry ratio, (**G**) leaf/stem ratio, and (**H**) specific leaf area. Data are means ± SE; *n* = 5. Error bars with different letters show a significant difference (*p* < 0.05).

Data statistics showed that with red–blue CL and red–blue–green CL, alfalfa growth traits exhibited different trends in response to nitrogen concentrations (Figure 2). The plant height, stem diameter, and leaf area of the red–blue CL were the highest in RB-CN_15_ and significantly higher than in treatments with low and high nitrogen concentrations (Figure 2A–C). The plant height and leaf area of alfalfa treated with red–blue–green CL were the highest under RBG-CN_20_, with no significant difference between the RBG-CN_15_ conditions, and significantly higher than samples treated with RBG-CN_10_. There was no significant difference in stem diameter between the RBG-CN_10_ and RBG-CN_15_ treatments. The plant height and leaf area of RBG-CN_10_ and RBG-CN_15_ treatments were significantly higher than those of plants treated with red–blue CL.

In terms of yield, fresh and dry yields with red–blue CL were the highest under the RB-CN_15_ treatment and significantly higher than the RB-CN_10_ and RB-CN_20_ treatments (Figure 2D,E). The fresh yield with RBG-CN_15_ and RBG-CN_20_ treatments was significantly higher than in alfalfa treated with RBG-CN_10_, while the dry yield with RBG-CN_20_ was the highest and significantly higher than the other five treatments. The leaf/stem ratio indicated that red–blue CL was higher than red–blue–green CL, with RB-CN_20_ being the highest (Figure 2F). The fresh/dry ratio of RBG-CN_15_ was the highest, which showed no significant difference compared to RBG-CN_20_ and was significantly higher than RBG-CN_10_. The lowest fresh/dry ratio was observed in RB-CN_20_ (Figure 2G). RB-CN_10_ and RBG-CN_15_ exhibited the lowest specific leaf areas (Figure 2H).

### 2.2. Photosynthetic Pigment Contents, Stomatal Conductance, and Fv/Fm of Leaves

**Figure 3:**
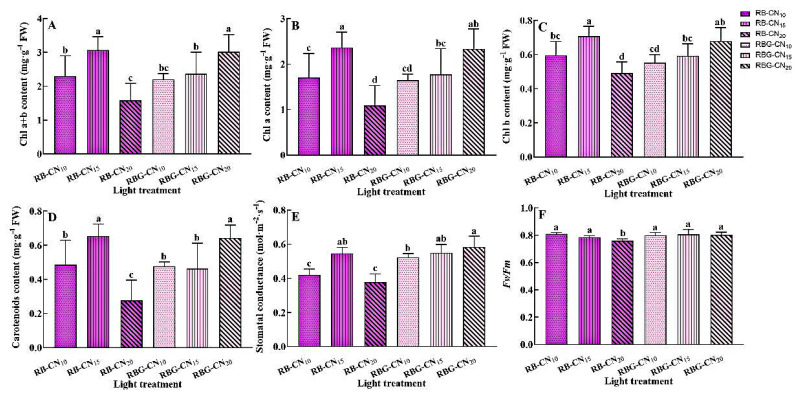
Photosynthetic pigment content, stomatal conductance, and *Fv*/*Fm* of alfalfa leaves under red and blue light and green light instead of red light. (**A**) Chl a + b content, (**B**) Chl a content, (**C**) Chl b content, (**D**) carotenoid content, (**E**) stomatal conductance, and (**F**) *Fv*/*Fm*. Data are means ± SE; *n* = 5. Error bars with different letters show a significant difference (*p* < 0.05).

The content of Chl a + b in RB-CN_15_ and RBG-CN_20_ was significantly higher than in other treatments. Specifically, the Chl a + b content in RB-CN_20_ was only 1.58 mg·g^−1^, which was 48.53% and 47.68% lower than in the RB-CN_15_ and RBG-CN_20_ treatments, respectively (Figure 3A). Chl a content was found to be the highest in the RBG-CN_15_ treatment and the lowest in the RB-CN_20_ treatment. The content of Chl a treated with RB-CN_20_ was only 1.09 mg·g^−1^, which was 53.81% and 53.42% lower than that of RB-CN_15_ and RBG-CN_20_, respectively (Figure 3B). The highest content of Chl b was found in RB-CN_15_, and the content of Chl b treated with RB-CN_20_ was reduced by 30.99% and 27.94% compared to RB-CN_15_ and RBG-CN_20_ treatments, respectively (Figure 3C). In terms of carotenoids, RB-CN_20_ treatment led to decreases of 57.58% and 56.25% compared to RB-CN_15_ and RBG-CN_20_ treatments, respectively (Figure 3D). There was no significant difference in the photosynthetic pigment content between RB-CN_10_, RBG-CN_10_, and RBG-CN_15_. Under the three nitrogen concentrations, the stomatal conductance of red–blue CL was 0.55–0.38 mol·m^−2^·s^−1^, and that of red–blue–green CL was 0.58–0.52 mol·m^−2^·s^−1^ (Figure 3E). The highest stomatal conductance was observed in the RBG-CN_20_ treatment, which was not significantly different from the RB-CN_15_ and RBG-CN_15_ treatments, but significantly higher than the RB-CN_10_, RB-CN_20_, and RBG-CN_10_ treatments. All treatments had *Fv*/*Fm* values above 0.75, and the RB-CN_20_ treatment led to significantly lower values than the other five treatments (Figure 3F).

### 2.3. Accumulation of Soluble Sugar, Sucrose, and Starch

**Figure 4:**
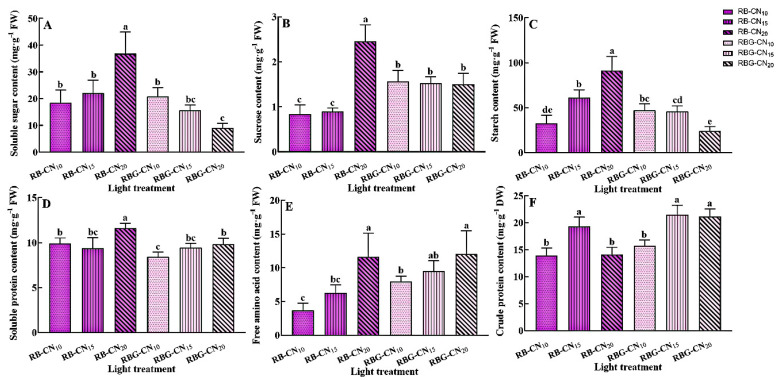
Soluble sugar (**A**), sucrose (**B**), starch (**C**), soluble protein (**D**), free amino acid (**E**), and crude protein (**F**) contents in alfalfa under three nitrogen concentrations and CL with two light qualities. Error bars with different letters show a significant difference (*p* < 0.05).

The soluble sugar content of red–blue CL increased with the increase in nitrogen concentration, while the soluble sugar content of red–blue–green CL decreased with the increase in nitrogen concentration (Figure 4A). The soluble sugar content of the RB-CN_20_ treatment was significantly higher than that of the other treatments, being 57.74% and 75.46% higher than the RBG-CN_15_ and RBG-CN_20_ treatments, respectively. The sucrose content of the RB-CN_20_ treatment was significantly higher than that of the other treatments. However, unlike soluble sugar, the sucrose content of the red–blue–green CL was higher than that of RB-CN_10_ and RB-CN_15_ (Figure 4B). Similarly, the starch content of RB-CN_20_ was significantly higher than in other treatments. With the increase in nitrogen concentration, the starch content of the red–blue CL increased, while the starch content of the red–blue–green CL significantly decreased with RBG-CN_20_ treatment (Figure 4C).

### 2.4. Accumulation of Soluble Protein, Free Amino Acids, and Crude Protein

The soluble protein content of RB-CN_20_ was significantly higher than that of the other treatments (Figure 4D). The RBG-CN_10_ treatment had the lowest soluble protein content, while there was no significant difference between the RB-CN_15_ and RBG-CN_15_ treatments. Both types of light resulted in higher soluble protein content under high nitrogen concentrations. Similar to soluble protein, free amino acids also showed an increase at high nitrogen concentrations, with RB-CN_20_ being significantly higher than the RB-CN_10_ and RB-CN_15_ treatments (Figure 4E) and RBG-CN_20_ only being significantly higher than RBG-CN_10_ (Figure 4F). The crude protein content of all treatments ranged from 13.93 to 21.50 mg·g^−1^, with higher levels observed in RB-CN_15_, RBG-CN_15_, and RBG-CN_20_, which were significantly higher than other treatments.

### 2.5. Antioxidant Activities

**Figure 5:**
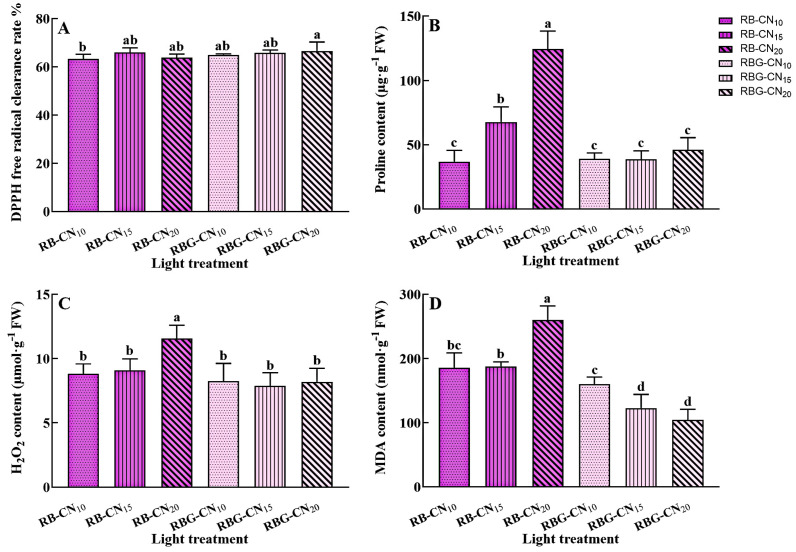
DPPH free radical clearance rate (**A**), Proline (**B**), H_2_O_2_ (**C**), and MDA (**D**) contents in alfalfa under three nitrogen concentrations and CL with two light qualities. Error bars with different letters show a significant difference (*p* < 0.05).

The DPPH free radical clearance rate of RBG-CN_20_ was significantly higher than that of the RB-CN_10_ treatment, and there were no significant differences among the other treatments (Figure 5A). The proline content of RB-CN_20_ was found to be the highest, increasing by 70.40% and 45.61% compared to the RBG-CN_10_ and RBG-CN_15_ treatments, respectively. The proline content of the red–blue CL significantly increased with the nitrogen concentration, while there was no significant difference in the proline content of the red–blue–green CL (Figure 5B). The H_2_O_2_ content of RBG-CN_20_ was significantly higher than in other treatments, and there was no significant difference in H_2_O_2_ content between the red–blue–green CL (Figure 5C). The highest MDA content was observed in RBG-CN_20_, which was significantly higher than in other treatments. Additionally, the MDA content of the red–blue–green CL was lower than that of the red–blue CL treatments (Figure 5D).

### 2.6. The Contents of Nitrate and Ammonium and Nr and Nir Activities

**Figure 6:**
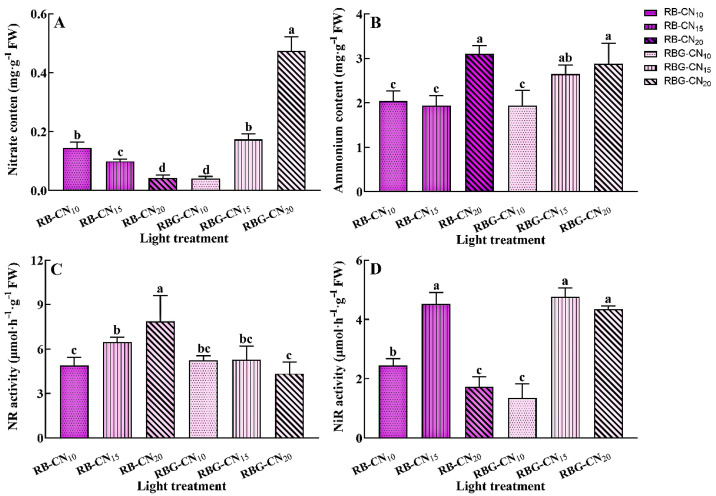
Nitrate (**A**) and ammonium (**B**) contents and activities of NR (**C**) and NiR (**D**) in alfalfa under three nitrogen concentrations and CL with two light qualities. Error bars with different letters show a significant difference (*p* < 0.05).

The response of nitrate content to nitrogen concentration of the two CLs was different (Figure 6A). The nitrate content of the red–blue CL decreased significantly with the increase in nitrogen concentration. The nitrate content of RB-CN_20_ was the lowest, being 72.43% and 60% lower than that of the RB-CN_10_ and RB-CN_15_ treatments, respectively. The nitrate content of red–blue and green CL increased with the rise in nitrogen concentration. The nitrate content of RBG-CN_20_ was the highest, being 76.47% and 91.49% higher than the RBG-CN_10_ and RBG-CN_15_ treatments, respectively. Although the nitrate content of RB-CN_20_ was lower, the ammonium content was the highest among all treatments under red–blue CL (Figure 6B), significantly exceeding the levels in other treatments. The ammonium content of red–blue–green CL also increased with the increase in nitrogen concentration. In terms of nitrogen metabolic enzyme activity, RB-CN_20_ exhibited the highest NR activity, which was significantly greater than that of other treatments, but the NiR activity was lower (Figure 6C,D). NR activity significantly increased with the rise in nitrogen concentration under red–blue CL. There was no significant difference in NR activity between red–blue–green CL treatments. The NiR activity of RB-CN_15_, RBG-CN_15_, and RBG-CN_20_ treatments was significantly higher than that of the other treatments.

### 2.7. The Contents of Glutamate, Cysteine, and Lysine and Their Metabolic Enzyme Activities

**Figure 7:**
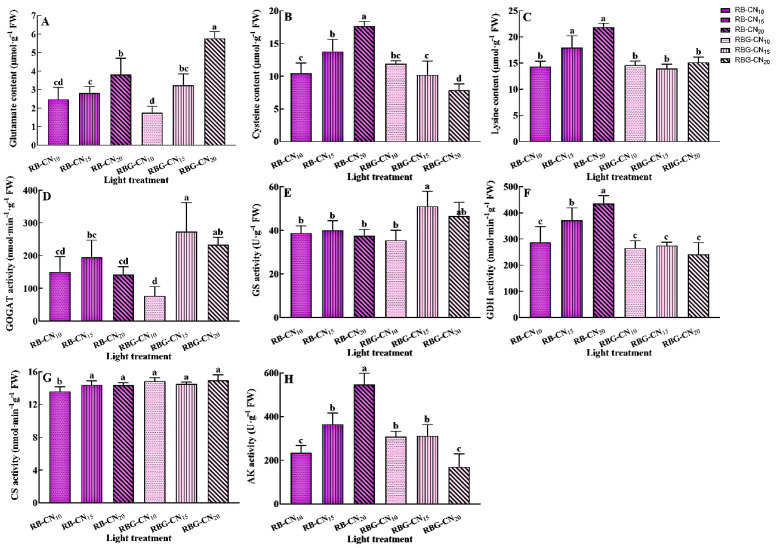
Glutamate (**A**), cysteine (**B**), and lysine (**C**) contents and activities of GOGAT (**D**), GS (**E**), GDH (**F**), CS (**G**), and AK (**H**) in alfalfa under three nitrogen concentrations and CL with two light qualities. Error bars with different letters show a significant difference (*p* < 0.05).

The glutamate content of both types of CL increased with the nitrogen concentration, and the glutamate content of red–blue–green CL showed a significant increase (Figure 7A). The glutamate content of RBG-CN_20_ was 69.67% higher than that of RBG-CN_10_ and 43.85% higher than that of RBG-CN_15_. The cysteine content in the red–blue CL significantly increased with the nitrogen concentration, whereas the cysteine content in the red–blue–green CL decreased as the nitrogen concentration increased (Figure 7B). The lysine content was higher in RB-CN10 and RB-CN_15_ treatments, significantly surpassing the levels found in the red–blue–green CL treatments (Figure 7C). There was no significant difference in the activities of GOGAT and GS under red–blue CL, but the activities of GOGAT and GS were significantly increased in RBG-CN_15_ and RBG-CN_20_ (Figure 7D,E). GDH activity under red–blue CL was significantly increased with increasing nitrogen concentrations, while there was no significant difference between treatments under red–blue–green CL (Figure 7F). The CS activity of RB-CN_10_ was significantly lower than that of other treatments, and the CS activity of the red–blue–green CL was not affected by nitrogen concentration (Figure 7G). The AK activity of the red–blue CL significantly increased with the nitrogen concentration, whereas the AK activity of the red–blue–green CL significantly decreased under the RBG-CN_20_ treatment (Figure 7H).

### 2.8. Nitrogen Concentration and Correlation with Parameters in CL

**Figure 8:**
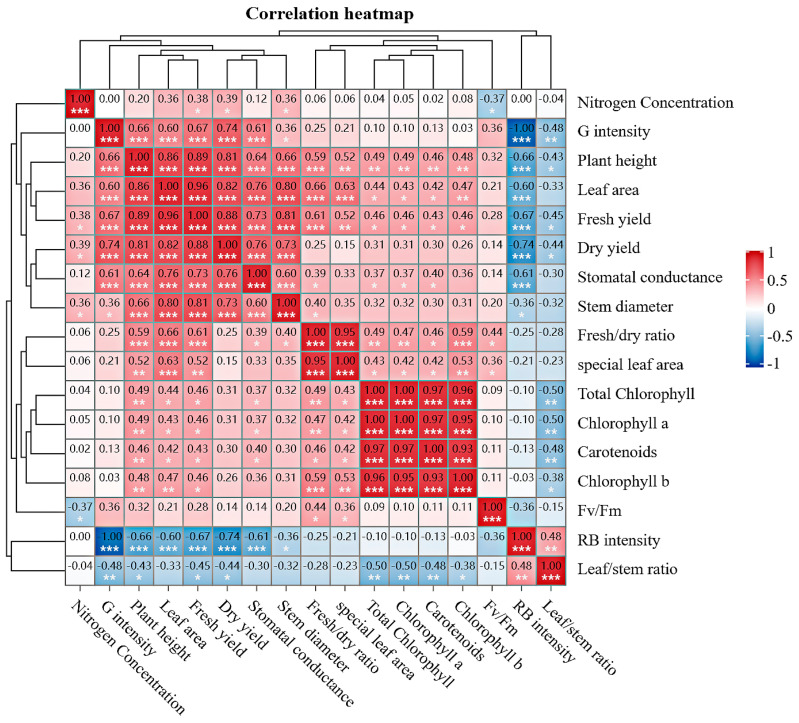
Correlation and heatmap analysis of growth characteristics, yield, photosynthetic pigment content, stomatal conductance, and *Fv*/*Fm* under three nitrogen concentrations and CL with two light qualities. Note. * Significant at the *p* < 0.05 level of probability, ** significant at the *p* < 0.01 level of probability, *** significant at the *p* < 0.001 level of probability.

Correlations between the nitrogen concentration and measured parameters under the two light quality treatments were analyzed (Figure 8). Nitrogen concentration was negatively correlated with *Fv*/*Fm* in alfalfa leaves, but not with growth traits and photosynthetic pigment content (Figure 8). The fresh yield, dry yield, stomatal conductance, leaf area, plant height, stem diameter, and *Fv*/*Fm* were positively correlated with an increase in green light intensity and a decrease in RB light intensity, while the leaf/stem ratio was negatively correlated. Nitrogen concentration under the two light quality treatments was positively correlated with plant height, leaf area, fresh yield, dry yield, fresh/dry ratio, specific leaf area, stomatal conductance, and photosynthetic pigment content (Figure 8). Under both types of light quality, the fresh yield of alfalfa was positively correlated with plant height, stem diameter, and leaf area. Fresh yield and stomatal conductance were positively correlated with photosynthetic pigment content.

### 2.9. Carbon and Nitrogen Substances Content and Correlation with Enzyme Activities in CL

**Figure 9:**
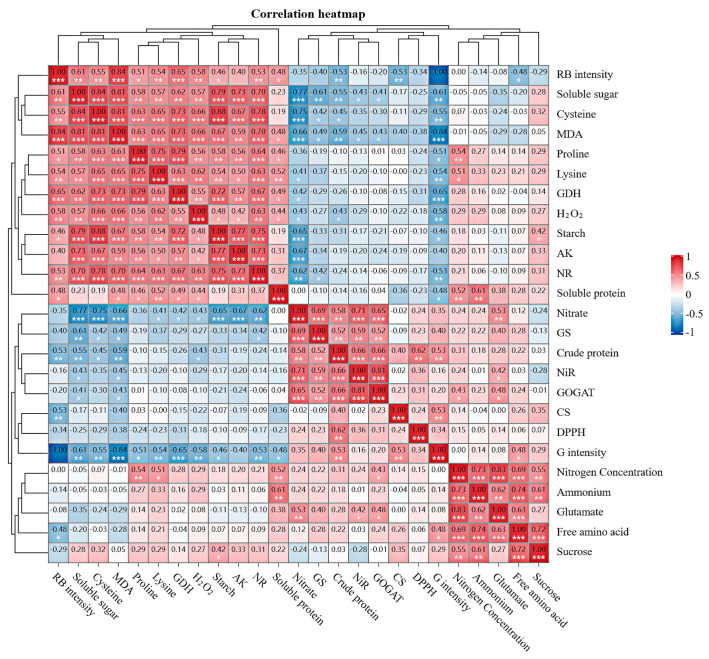
Correlation and heatmap analysis of carbon and nitrogen substances and enzyme activities under three nitrogen concentrations and CL with two light qualities. Note. * Significant at the *p* < 0.05 level of probability, ** significant at the *p* < 0.01 level of probability, *** significant at the *p* < 0.001 level of probability.

There were varying degrees of correlation between physiological indexes of alfalfa under two types of light quality and three levels of nitrogen concentrations (Figure 9). Under two types of light quality, soluble sugar was positively correlated with cysteine, MDA, starch, sucrose, proline, lysine, H_2_O_2_, and the activities of NR, GDH, and AK. The levels of crude protein, GS, and free amino acids were positively correlated with an increase in green light intensity and a decrease in RB light intensity, while MDA, soluble sugar, cysteine, lysine, H_2_O_2_, proline, starch, soluble protein, and the activities of NR and GDH were negatively correlated. The contents of soluble protein were positively correlated with ammonium, lysine, MDA, proline, and H_2_O_2_. The crude protein content was positively correlated with NiR, GOGAT, nitrate, GS, and the DPPH free radical clearance rate. The nitrate content was negatively correlated with carbohydrate content, proline, MDA, H_2_O_2,_ and the activities of NR, GDH, and AK (Figure 9). No correlation was found between soluble sugar and nitrate. Starch content was negatively correlated with nitrate, crude protein, GS, NiR, and GOGAT, but positively correlated with carbohydrates, cysteine, MDA, proline, H_2_O_2_, and the activities of NR, GDH, and AK (Figure 9). The contents of glutamate, ammonium, free amino acids, sucrose, proline, lysine, and GOGAT under CL with two light qualities were positively correlated with the nitrogen concentration (Figure 9).

### 2.10. The Interaction Between Nitrogen Concentration and Light Quality on Indicators in Alfalfa

The interaction between nitrogen concentration and light quality on various indicators of alfalfa was analyzed. Two-factor analysis of variance showed that the interaction between nitrogen concentration and light quality significantly affected the growth of alfalfa, including plant height, stem diameter, leaf area, fresh yield, dry yield, fresh/dry ratio, and stomatal conductance, as well as the contents of total chlorophyll, chlorophyll a, chlorophyll b, and carotenoids (*p* < 0.001) (Table 1). Meanwhile, soluble sugar, sucrose, starch, soluble protein, free amino acids, crude protein, and proline were significantly influenced by the interaction between the nitrogen concentration and light quality (*p* < 0.001) (Table 2). In addition, the contents of MDA, H_2_O_2_, nitrate, ammonium, NR, NiR, glutamate, cysteine, and lysine, as well as the activity of GOGAT, GDH, and AK, in alfalfa were significantly affected by this interaction (*p* < 0.001) (Table 2). The interaction had significant effects on special leaf area, *Fv*/*Fm*, and GS activities (*p* < 0.01) (Table 2). Similarly, the activity of CS was significantly affected by this interaction (*p* < 0.05). The interaction of nitrogen concentration and light quality had no effect on the leaf/stem ratio and DPPH (Table 1 and Table 2).

## 3. Discussion

The nitrogen concentration in the nutrient solution plays a crucial role in regulating plant growth and development. Sun et al., 2023 found that the forms and proportions of nitrogen had notable impacts on plant height, stem diameter, and leaf area [30]. Appropriate nitrogen application was shown to significantly enhance various growth parameters in tomato plants. In this study, we observed that nitrogen concentrations significantly affected the stem growth of alfalfa in terms of plant height and stem diameter. The lowest nitrogen dose appeared insufficient to support growth, while high nitrogen inhibited stem elongation under red–blue CL but had no significant effect under red–blue–green CL. Combined with the morphology of leaf chlorosis, it was concluded that the stress injury was caused by high nitrogen treatment under red–blue CL. Under the same nitrogen concentration, the leaf area of red–blue–green CL is always higher than red–blue CL, which is consistent with previous findings that green light can promote leaf area expansion [14]. Xie et al., 2015 conducted an experiment with three nitrogen application treatments on alfalfa (0, 75, and 150 kg·ha^−1^) [31]. They calculated the yield from three harvests and found that nitrogen application did not significantly enhance the yield. Kamran et al., 2022 found that under the same irrigation conditions, nitrogen application of 150 kg·ha^−1^ can significantly increase the yield compared with no nitrogen application [5]. However, when nitrogen application is increased to 300 kg·ha^−1^, the yield decreases. An appropriate nitrogen concentration promotes root activity and the nitrogen use efficiency of root nodules, enhancing resource acquisitions that regulate the distribution of photo-assimilates in the shoot of the plant [32]. The influence of nitrogen fertilizer on yield is related to irrigation, variety characteristics, basic soil nutrients, and nodule nitrogen fixation, among other factors. Combined with the fact that under the same high nitrogen level of red–blue–green CL in this study, alfalfa leaf area and hay yield increased but did not exhibit the same high-nitrogen inhibition effect as that of red–blue CL, this suggests that light composition and light pattern are also crucial environmental factors for high nitrogen adaptation.

The leaf area of red–blue CL treated with high levels of nitrogen was lower, but the leaf/stem ratio was significantly higher than samples treated with red–blue–green CL. Additionally, the fresh/dry ratio was lower, possibly due to the high dry weight of leaves resulting from the accumulation of large amounts of starch in the leaves. There was a significant difference in leaf area among the red–blue–green CL, but no significant difference in the leaf/stem ratio. Although significantly lower than red–blue CL samples treated with high levels of nitrogen, the leaf/stem ratio can reach 0.7–0.89 when cultivated in the field [33]. Studies have shown that nitrogen addition is positively correlated with the specific leaf area of plants [1]. In this experiment, there was no correlation between specific leaf area and nitrogen concentration under CL with two light qualities. The results of this study showed that alfalfa was damaged by stress under the high-nitrogen treatment of red–blue CL, while the yield of alfalfa increased under the high-nitrogen water treatment of red–blue–green CL. This increase was attributed to the expansion of leaf area, higher photosynthetic pigment content, and improved photosynthetic capacity induced by green light, resulting in greater biomass accumulation.

The chlorophyll content was consistent with the color of alfalfa leaves. The leaves turned chlorotic under RB-CN_20_ treatment, and the photosynthetic pigment content decreased significantly. Nitrogen is crucial for chlorophyll synthesis. Wu et al., 2019 found that the content of chlorophyll a decreased significantly under a low-nitrogen environment, and the results of this study were consistent with this under the two light qualities [34]. Under a high-nitrogen environment, the photosynthetic pigment content of red–blue CL decreased significantly, while that of red–blue–green CL continued to increase. This phenomenon may be attributed to the fact that green light can penetrate deeper into leaf tissues, enhancing photosynthesis in deeper cell layers [35,36]. In addition, Bian et al., 2018 found that adding green light to red–blue light could reduce the negative effects of continuous light (CL) on lettuce photosynthesis and improve *Fv*/*Fm* [23]. In this study, the stomatal conductance of red–blue–green CL with high nitrogen was also significantly higher than that of the red–blue CL treatment. *Fv*/*Fm* values, typically ranging between 0.75 and 0.85, have been utilized as a reliable indicator for assessing abiotic stress on PSII [37]. Although the lower leaves of the red–blue CL high-nitrogen samples showed signs of stress damage, the *Fv*/*Fm* values of the upper mature leaves remained above 0.75. However, they were notably lower compared to the *Fv*/*Fm* values of the other treatments. The photosynthetic pigment content, stomatal conductance, and *Fv*/*Fm* values of red–blue–green CL with high nitrogen did not decrease. This suggests that red–blue–green CL enhances the adaptability of the plant’s photosynthetic capacity to high nitrogen levels compared to red–blue CL.

Nitrogen application can affect plant photosynthesis and regulate the distribution of carbohydrates between source and sink. In general, the content of soluble sugar and starch increases within the optimal nitrogen application range. This increase is directly proportional to the nitrogen levels. Moreover, the distribution of carbohydrates is influenced by the growth stage [38,39]. In this study, carbohydrate accumulation and distribution under different nitrogen concentrations were also influenced by light quality. Nitrogen levels were positively correlated with soluble protein, crude protein, and starch, while soluble sugar was negatively correlated with nitrogen levels. In most plants, starch is produced in the chloroplast through photosynthesis and is utilized during the night [40]. The starch content accumulated significantly under the treatment of high nitrogen in red–blue CL. On the one hand, there was no dark environment under CL conditions to generate starch. On the other hand, the plants grew short and developed slowly, which reduced starch consumption. The contents of soluble sugar and starch in red–blue–green CL with high nitrogen were lower compared to other treatments, but their dry weight was the highest. It is speculated that soluble sugar and starch were utilized in the synthesis of structural carbohydrates, leading to the accumulation of more polymer compounds, such as cellulose, in alfalfa.

The nitrogen levels were positively correlated with sucrose, soluble protein, proline, and free amino acids, and the content of free amino acids was positively correlated with the content of ammonium, sucrose, and glutamate. With the increase in nitrogen levels, the accumulation of ammonium in alfalfa increased, thereby promoting the synthesis of amino acids. For legumes, excessive nitrogen fertilizer application reduces the number of nodules and nitrogen fixation ability, as shown by Sanginga [41], thus affecting the synthesis of crude protein. Zhang et al., 2020 harvested alfalfa for two years, cutting it four times a year, and analyzed the changes in crude protein content under three nitrogen levels (0, 105, and 210 kg·ha^−2^) [27]. They found that the crude protein content was generally higher at 105 kg·ha^−2^. Zhao et al., 2023 conducted a study applying two nitrogen levels and four phosphorus levels to alfalfa, resulting in a total of eight fertilization treatments [39].

They discovered that when the nitrogen fertilizer reached a certain threshold, applying a specific amount of phosphorus fertilizer could further enhance the yield and feeding quality of alfalfa. In order to further increase the crude protein content, changing the nitrogen level in CL can affect the supply of phosphorus. Proline plays a crucial role in stabilizing structural components and enzyme structures, as well as regulating osmotic pressure to withstand abiotic stresses such as light, high salt levels, and high temperatures [42,43]. At the same time, as an amino acid synthesized with glutamate or ornithine as a substrate, its content is also affected by nitrogen supply. Nasab et al., 2014 found that increasing nitrogen application could enhance the proline content and reduce the reducing sugar content in pistachio (*Pistacia vera* L.) seedlings [44]. Additionally, the performance of red–blue–green CL was similar. On the one hand, the high nitrogen content of red–blue CL resulted in increased glutamate production, which facilitated proline synthesis. On the other hand, the stress caused alfalfa to accumulate proline.

Light quality affects the absorption and utilization of nitrate by plants [45]. Chen et al., 2024 found that red–blue–green light was more conducive to the absorption and accumulation of nitrate by alfalfa compared to red–blue light [14]. In this study, there was a significant difference in the nitrate content of the two types of CL. The nitrate content of red–blue CL decreased at high nitrogen concentrations, whereas the nitrate content of red–blue–green CL increased. However, the ammonium content exhibited relative consistency, and correlation analysis revealed that the ammonium content increased with the rise in nitrogen concentration under both light qualities. Plant adaptability to ammonium content is affected by genetic characteristics, which not only vary among species but also significantly among different varieties of the same species [46]. High ammonium content can inhibit plant growth and lead to toxicity accumulation in cells [47]. The high ammonium content in red–blue CL, along with the excessive accumulation of H_2_O_2_ and MDA, all indicate that the slow chlorosis of leaves under this treatment is caused by ammonium toxicity. Xiao et al., 2023 reviewed various methods to inhibit ammonium toxicity, such as inhibiting ammonium transport, removing reactive oxygen species, and utilizing potassium [48]. We suggest that incorporating green light into the light spectrum could also be a promising approach to mitigate ammonium toxicity.

Nitrogen accumulation is closely related to the activity of enzymes involved in nitrogen metabolism. Suitable nitrogen application can increase the activity of enzymes related to nitrogen metabolism, enhancing the metabolism and synthesis of nitrogen-containing compounds in leaves. NR activity is induced and regulated by light, nitrate, sugar, and organic acids [49]. Under CL with two light qualities, NR activity was positively correlated with concentrations of soluble sugar, sucrose, and nitrogen. On the one hand, the NR reaction depletes ATP, and the large amount of accumulated carbohydrates can provide sufficient energy for the reaction. On the other hand, it is possible that sugar can replace light to induce NR activity to remain at a high level [50]. Wang et al., 2000 found that a high nitrate content would lead to the reduced expression of genes regulating NR and inhibited transporters [51]. Under red–blue–green CL, the levels of soluble sugar and nitrate accumulation significantly reduced the activity of NR. Although the NiR activity of red–blue CL treatment with high nitrogen was significantly reduced, the activity of NR was higher, thus maintaining the ammonium content at a high level.

Glutamate content exhibited a similar positive correlation trend with nitrogen concentration under both light qualities, but cysteine showed differences. Glutamate and glutamine are produced through nitrogen assimilation through the GOGAT/GS cycle. The glutamate content was positively correlated with the ammonium content under both light conditions. The higher content of ammonium provided sufficient substrates for the synthesis of glutamate. Research results on glutamate-metabolizing enzyme activity vary with nitrogen application levels. Wang et al., 2018 analyzed the nitrogen metabolism of rice under four nitrogen levels (0–300 kg·ha^−1^) during the stages of germination, heading, and grain filling [52]. They found that GS activity increased with a higher nitrogen application rate, while GOGAT and GDH decreased. Song et al., 2023 analyzed the activities of GOGAT, GS, and GDH in rice leaves from earing to maturity under six nitrogen application levels (0–400 kg·ha^−1^) [53]. They found that the enzyme activities initially increased and then decreased with the nitrogen level, reaching the highest level at a nitrogen application rate of 320 kg·ha^−1^. The application of 400 kg·ha^−1^ of nitrogen inhibited enzyme activity and led to an excessive accumulation of ammonium. In this study, the activities of GOGAT and GS initially increased and then decreased with the nitrogen level under red–blue CL. However, they only increased under red–blue–green CL. This suggests that green light raised the threshold for alfalfa’s adaptation to liquid nutrient nitrogen concentrations. The higher GOGAT activity under red–blue–green light and high nitrogen treatment resulted in increased glutamate synthesis. It is worth noting that the activity of GDH is significantly increased under high nitrogen concentrations in red–blue CL. It was reported that the decline in GS/GOGAT activities under salinity stress restricts glutamine and amino acid production while promoting NH^4+^ accumulation, thereby activating the GDH pathway [54], which is consistent with the results of this study.

Chen et al., 2024 found that replacing part of the red light with green light in red–blue light would reduce the content of cysteine and lysine [14]. In this study, the same phenomenon occurred when the nitrogen concentration exceeded 10 mM. CS can catalyze the reaction of OAS and H_2_S to produce cysteine [55], and the key to cysteine synthesis is the source of the thiohydric group. In this study, no correlation was found between cysteine content and CS activity. Further analysis of the response of cysteine to nitrogen levels should be conducted by detecting the sulfur element. Rao et al., 1999 found that the activity of AK was significantly regulated by the light quality [56]. Interestingly, in this study, AK activity was positively correlated with nitrogen levels under CL with two qualities.

## 4. Materials and Methods

### 4.1. Plant Material and Growth Conditions

*M. sativa* (Gannong No. 3) was designated as the experimental plant species, with the procurement of seeds from the commercial market. The experiment was conducted at PFAL located in the Institute of Environment and Sustainable Development in Agriculture, Chinese Academy of Agricultural Sciences. Throughout the course of the experiment, the internal temperature of the PFAL was maintained at 24 ± 2 °C, while the relative humidity ranged between 35% and 50%. The concentration of CO_2_ was maintained at 450 ± 50 ppm.

The composite light source consisted of red–blue light LEDs (red light peak at 655 nm, blue light peak at 437 nm) and red–blue–green light LEDs (red light peak at 662 nm, blue light peak at 460 nm, and green light peak at 520 nm) on a combination lamp board (Wuxi Huazhaohong Optoelectronics Technology Co. Ltd., Wuxi, China) measuring 50 cm × 50 cm. The LED panel lights were suspended at a height of 40 cm above the plug trays, and the light intensity on the surface of the plug trays was measured and then designated as the experimental light intensity. The plug trays measure 54 cm × 28 cm and contain 32 cells.

Solid medium was filled in the cell with a plug tray made of a mixture of perlite, vermiculite, and turfy soil in a 1:1:3 ratio. Then, distilled water was added and the water content was adjusted to 55–60% of the substrate. Pile for 2–3 h until the matrix has absorbed the water sufficiently, then pack evenly in the hole dishes [13]. Seven full seeds of uniform size are sown at each cell. The seedlings were thinned to a density of five plants per cell when the plant’s first leaf was completely expanded. The detailed description of the experimental protocol can be seen in our previous research [13,14].

### 4.2. Light and Nitrogen Concentration Treatments

Red–blue (RB) and red–blue–green (RBG) light treatments were applied continuously with a Daily Light Integral (DLI) of 23.328 μmol·m^−2^·d^−1^. The RB treatment consisted of a red light intensity of 216 μmol·m^−2^·s^−1^ and blue light at 54 μmol·m^−2^·s^−1^. The RBG treatment included red light at 210 μmol·m^−2^·s^−1^, blue light at 52.5 μmol·m^−2^·s^−1^, and green light at 7.5 μmol·m^−2^·s^−1^. Alfalfa was cultured in Hoagland solution containing 4 mM Ca(NO_3_)_2_·4H_2_O, 6 mM KNO_3_, 1 mM NH_4_H_2_PO_4_, 2 mM MgSO_4_·7H_2_O, 71 μM Fe-EDTA-Na_2_, 46 μM H_3_BO_3_, 9.6 μM MnSO_4_·4H_2_O, 0.07 μM ZnSO_4_·7H_2_O, 0.3 μM CuSO_4_·5H_2_O, and 0.07 μM (NH_4_)_6_Mo_7_O_24_·4H_2_O. The plants were cultured in this solution until they had grown their first true leaves. The nutrient solution with NO_3_^–^ concentrations of 10, 15, and 20 mM was prepared by adjusting the KNO_3_ content and was labeled as N10, N_15_, and N20, respectively. The seed was planted on 27 July 2023 and harvested after 30 days [13].

### 4.3. Determination of Agronomic Traits and Yield

Five plant samples were randomly selected and harvested on the 30th day while maintaining a consistent stubble height of 10 ± 0.5 cm. Vernier calipers and straighteners were used to measure the cut alfalfa. The thickness of the stem base was used as an indicator of stem diameter, while the distance from the base to the growing point was measured as plant height. Furthermore, the fresh yield was weighed using an electronic balance. The leaves were then cut, and the leaf area of alfalfa leaves was measured using the LI-3100 C leaf area meter (LI-COR Biosciences, Lincoln, NE, USA). For measuring dry weight, plant materials were dried at 70 °C for 72 h. Subsequently, the leaf/stem ratio, fresh/dry ratio, and specific leaf area were calculated.

### 4.4. Determination of Photosynthetic Pigment Content

For calculation of total chlorophyll (Chl a + b), chlorophyll a (Chl a), chlorophyll b (Chl b), and carotenoid contents, the third mature leaf at the tip was gathered. Photosynthetic pigment contents were analyzed using the method adapted from Lichtenthaler and Wellburn [57]. In short, photosynthetic pigments were determined using a 0.1 g cutting sample, which was placed in a 10 mL centrifuge tube containing 5 mL of 95% ethanol solution. The centrifuge tube was wrapped with aluminum foil and extracted in the dark at room temperature for 48 h [58]. The absorbance of the solution was measured with a microplate reader (TECAN, Infinite M200 Pro, Männedorf, Switzerland) at 663, 646 nm, and 470 nm.

### 4.5. Determination of Stomatal Conductance and Fv/Fm

For operation, the third mature leaf at the tip of the alfalfa was selected for measurement. The stomatal conductance and *Fv*/*Fm* of alfalfa leaves were measured using a porometer/fluorometer LI-600 (LI-COR Biosciences, Lincoln, NE, USA). Before fluorometer measurements, the whole plant was dark-adapted for 30 min.

### 4.6. Measurement of Plant Physiological Indicators

Plants from four cells were randomly selected while maintaining a consistent stubble height of 10 ± 0.5 cm. The samples were packed with aluminized paper and stored in a refrigerator at −80 °C after being treated with liquid nitrogen. Subsequently, the samples used for the determination of the ingredient indices were thoroughly powdered with liquid nitrogen using a mortar and pestle.

Sucrose (Cas No.: BC2465), starch (Cas No.: BC0705), free amino acids (Cas No.: BC1575), proline (Cas No.: BC0295), ammonium (Cas No.: BC1525), glutamate (Cas No.: BC1585), and cysteine (Cas No.: BC0185) were quantitatively assessed using the corresponding kits (Beijing Solarbio Science & Technology Co., Ltd., Beijing, China) through spectrophotometric measurements in accordance with the manufacturer’s instructions. The content of lysine was quantitatively assessed using the corresponding kit (Suzhou Michy Biomedical Technology Co., Ltd., Suzhou, China, M0508A).

Soluble sugar, soluble protein, and nitrate contents were also determined using fresh powdered samples. The quantification of soluble sugar content was conducted using the phenol-sulfuric acid method [59]. The determination of soluble protein content was accomplished through the utilization of the Coomassie brilliant blue method [60]. The content of nitrate was assessed employing the salicylic acid method [61].

Dried leaves and stems were mixed and fully ground using a high-throughput tissue grinder (SCIENTZ-48, Ningbo Scientz Biotechnology Co., Ltd., Ningbo, China). The crude protein content of alfalfa was measured by the Kjeldahl method using an ATC-206 tester (KDY-9820, Beijing tongrunyuan electromechanical technology Co., Ltd., Ningbo, China). Crude protein was calculated by multiplying the determined nitrogen value by 6.25.

### 4.7. Measurement of Enzyme Activity

The activities of NR (Cas No.: BC0085), NiR (Cas No.: BC1545), GS (Cas No.: BC0915), GOGAT (Cas No.: BC0075), and GDH (Cas No.: BC1465) were assessed spectrophotometrically employing the corresponding kits (Beijing Solarbio Science & Technology Co., Ltd., Beijing, China) through spectrophotometric measurements in accordance with the manufacturer’s instructions.

The activity of CS was measured in accordance with the reference methodology [62], wherein it was expressed as 1 nmol OAS per minute in 1 g of fresh tissue. AK activity was determined following the established procedures outlined by Brennecke [63]. The activity of AK was expressed as follows: a change of 0.005 light absorption per hour at 505 nm was defined as one unit of activity.

### 4.8. Statistical Analysis

The data were analyzed using SPSS 23.0 (SPSS, Inc., Chicago, IL, USA). The interaction between nitrogen concentration and light quality on the parameters of alfalfa was analyzed by two-way ANOVA analysis, while other data were subjected to one-way ANOVA analysis. Significant differences among treatment means were determined using the LSD test at a 95% confidence level. The column diagrams were all created using GraphPad Prism v8.0 (GraphPad Software Inc., San Diego, CA, USA). Correlation analysis heat maps were generated using the OmicShare tools.

## 5. Conclusions

There were significant differences in the response of alfalfa to nitrogen concentrations under two different light qualities. The fresh yield, dry yield, stomatal conductance, leaf area, plant height, stem diameter, crude protein, GS, and free amino acids of alfalfa were positively correlated with the increase in green light intensity, thereby improving the yield and quality of alfalfa. With the increase in nitrogen concentration, photosynthetic capacity, NiR, and GOGAT activities increased, promoting growth and enhancing feeding value. In conclusion, nitrogen application could promote the growth and quality of alfalfa. The interaction between nitrogen concentration and light quality significantly affected the growth, yield, photosynthetic pigments, carbon, nitrogen substances, and enzyme activities of alfalfa, and had no effect on the leaf/stem ratio and DPPH. Additionally, the inclusion of green light can enhance alfalfa’s tolerance to varying concentrations of liquid nitrogen nutrients. The optimal nitrogen concentration under red–blue CL is 15 mM, while under red–blue–green CL, it is 20 mM. These research results can be used to optimize environmental parameters in PFAL to maximize the yields and commercial values of forage production.

## Figures and Tables

**Table 1:** The interaction between nitrogen concentration and light quality on the indicators of growth, yield, and photosynthetic pigments in alfalfa.

Indicators	Plant Height	Stem Diameter	Leaf Area	Fresh Yield	Dry Yield	Leaf/Stem Ratio	Fresh/Dry Ratio	Special Leaf Area	Total Chlorophyll	Chlorophyll a	Chlorophyll b	Carotenoids	Stomatal Conductance	Fv/Fm
Nitrogen concentration × light quality	***	***	***	***	***	/	***	**	***	***	***	***	***	**

Note. ** significant at the *p* < 0.01 level of probability, *** significant at the *p* < 0.001 level of probability.

**Table 2:** The interaction between nitrogen concentration and light quality on the indicators of carbon, nitrogen substances, and enzyme activities in alfalfa.

Indicators	Soluble Sugar	Sucrose	Starch	Soluble Protein	Free Amino Acid	Crude Protein	Proline	DPPH	MDA	H_2_O_2_	Nitrate	Ammonium	NR	NiR	Glutamate	Cysteine	Lysine	GOGAT	GS	GDH	CS	AK
Nitrogen concentration × light quality	***	***	***	***	***	***	***	/	***	***	***	***	***	***	***	***	***	***	**	***	*	***

Note. * Significant at the *p* < 0.05 level of probability, ** significant at the *p* < 0.01 level of probability, *** significant at the *p* < 0.001 level of probability.

## Data Availability

Please contact the corresponding author for any additional information.
